# Scanning Hyperspectral Imaging for In Situ Biogeochemical Analysis of Lake Sediment Cores: Review of Recent Developments

**DOI:** 10.3390/jimaging8030058

**Published:** 2022-02-25

**Authors:** Paul D. Zander, Giulia Wienhues, Martin Grosjean

**Affiliations:** Oeschger Centre for Climate Change Research and Institute of Geography, University of Bern, 3012 Bern, Switzerland; giulia.wienhues@giub.unibe.ch (G.W.); martin.grosjean@oeschger.unibe.ch (M.G.)

**Keywords:** hyperspectral imaging, VNIR, image classification, reflectance spectroscopy, paleolimnology, lake sediments, sedimentary pigments, environmental change

## Abstract

Hyperspectral imaging (HSI) in situ core scanning has emerged as a valuable and novel tool for rapid and non-destructive biogeochemical analysis of lake sediment cores. Variations in sediment composition can be assessed directly from fresh sediment surfaces at ultra-high-resolution (40–300 μm measurement resolution) based on spectral profiles of light reflected from sediments in visible, near infrared, and short-wave infrared wavelengths (400–2500 nm). Here, we review recent methodological developments in this new and growing field of research, as well as applications of this technique for paleoclimate and paleoenvironmental studies. Hyperspectral imaging of sediment cores has been demonstrated to effectively track variations in sedimentary pigments, organic matter, grain size, minerogenic components, and other sedimentary features. These biogeochemical variables record information about past climatic conditions, paleoproductivity, past hypolimnetic anoxia, aeolian input, volcanic eruptions, earthquake and flood frequencies, and other variables of environmental relevance. HSI has been applied to study seasonal and inter-annual environmental variability as recorded in individual varves (annually laminated sediments) or to study sedimentary records covering long glacial–interglacial time-scales (>10,000 years).

## 1. Introduction

The scientific investigation of sediment deposited in lakes can provide important and diverse information about past changes in environmental conditions, as well as human impacts to the natural environment, over timescales ranging from the recent past to over 100,000 years [1,2]. Lake sediments are highly valued by geoscientists because they record a history of environmental changes in the atmosphere, cryosphere, hydrosphere, lithosphere, and biosphere. In recent years, non-destructive sediment core-scanning techniques (e.g., micro X-ray fluorescence, X-ray radiography, p-wave velocity and gamma density, computed tomography, and magnetic susceptibility) have become widely used in earth sciences due to their ability to obtain high-resolution data quickly, while preserving sample material for further wet-chemical or physical analyses [3,4,5]. Hyperspectral imaging (HSI) core scanning is a recently developed technique in which a hyperspectral camera records light spectra reflected directly from the surface of a sediment core. Each pixel within the hyperspectral image contains a spectral reflectance profile that can be used to gain information about the characteristics of the sediment within that pixel [6]. Using this approach, physical, mineralogical, and biogeochemical data can be acquired at submillimeter spatial resolution (40–300 μm).

This ultra-high measurement resolution is especially valuable in studies of lake sediments, because spatial resolution, along with sedimentation rate, determines the temporal resolution of the data obtained from sediments. To convert sediment depths into ages and determine sedimentation rates, a variety of geochronological techniques can be used, including radiocarbon dating [7] and varve (annual layer) counting [8]. Typical sedimentation rates range from 0.1 to 10 mm per year. Conventional analyses of sediments require destructive sampling of the sediment core, which typically requires samples to be greater than 5 mm thick. Therefore, HSI data can increase the temporal resolution of data generated from sediment cores by 1–2 orders of magnitude compared to conventional analyses, making it possible to obtain data at sub-annual resolution. An additional advantage of HSI in this field is that spatial information about sediment properties can be obtained. This is not possible with other point-based spectroscopy techniques [9,10], and this spatial information is useful for paleoclimate and paleoenvironmental interpretations.

HSI of lake sediment cores is a novel technique rapidly growing in interest, particularly due to its rapid acquisition of ultra-high-resolution datasets. HSI core scanning has utilized cameras operating in the visible and near infrared (VNIR; 400–1000 nm) and short wave infrared (SWIR; 1000–2500 nm) wavelengths to detect sedimentary components and properties such as organic matter [11], sedimentary pigments [12,13,14], minerals [12], particle size [15,16], and sedimentary structures [17,18].

In this review, we summarize the state-of-the-art application of HSI to lake sediment cores. We present the development of the technique from VNIR reflectance spectroscopy to HSI core-scanning. Then, we describe the available equipment and general methodological workflow used for in situ HSI core scanning and data processing. Next, we review common biogeochemical interpretations of sediment reflectance spectra. The potential and limitations of the HSI core-scanning method are discussed. Finally, we assess the peer-reviewed literature on HSI core scanning of lake sediments and present several key research themes where the application of HSI has led to important insights relevant for paleoenvironmental research.

## 2. Development of the Hyperspectral Imaging Core-Scanning Technique

It has been recognized for a long time that sediment color in the visible (VIS) range (i.e., what we see with our eyes) contains valuable information about organic and inorganic sediment components, lithotypes, and depositional environments. The pioneering work of Swain [19], Züllig [20], and others has revealed that (1) colored sedimentary pigments, such as chlorophylls and carotenoids, are well preserved in lake sediments, and (2) pigments contain diagnostic information about past environmental conditions such as paleoproductivity, past anoxia events, and potentially harmful cyanobacterial algal blooms. As early as the 1980s, these insights sparked interest in developing non-destructive, rapid, labor- and cost-efficient techniques to measure pigments, organic matter, and minerals in lake and marine sediments.

Deaton and Balsam [21] provided the proof of concept that visible reflectance spectroscopy (VIS-RS; 400–700 nm) is a suitable rapid and non-destructive technique to detect iron oxides (hematite and goethite) in marine sediments. Iron oxides in North Atlantic sediments are highly relevant because they preserve information about the source areas of the sediment and ocean currents in the past. Balsam and Deaton [22] extended reflectance spectrophotometry to the ultraviolet (UV) and near infrared (NIR) ranges, which allowed them to diagnose carbonate, organic matter, opal, and clay minerals. Interestingly, they were already able to take point measurements directly from the fresh sediment cores with a measurement field of 2 cm diameter.

A major innovation was brought forward by Rein and Sirocko [9]. They used a low-cost handheld GretagMacbeth Spectolino (380–730 nm, spectral resolution 10 nm) with a measurement field of 2 mm and developed algorithms for spectral indices that were calibrated against chlorophyll *a* and derivatives (index: relative absorption band depth between 660 and 670 nm; RABD_660;670_), carotenoids (index: RABD_510_), and lithogenic components (mainly illite, chlorite, and mica; ratio between reflectance at 570 nm and 630 nm, index: R_570_/R_630_). Spectrolino point measurements were taken directly from the fresh sediment surface (in situ) at a resolution of 2 mm. In a case study on a marine core from offshore Peru, Rein et al. [23] used the aforementioned spectral indices to establish a 20,000-year-long reconstruction of ENSO (El Niño–Southern Oscillation) events at an unprecedented temporal resolution.

The application of VIS-RS techniques in lake sediments lagged behind the developments of the marine sediment community. Das et al. [24] and Wolfe et al. [25] established that sediment reflectance around 675 nm (in bands 650–700 nm) can be calibrated to sedimentary chlorophyll *a* and related derivatives, which is useful to track past trophic states in lakes. However, they used a FieldSpec Pro spectroradiometer (350–2500 nm) which required subsampling, freeze drying, and the homogenization of sediments; therefore, this limited the number (and resolution) of measurement points that could be processed. Michelutti et al. [26] and Michelutti and Smol [27] demonstrated that chlorophyll concentrations could be estimated using this technique in a wide variety of environments from arctic to tropical lakes. Ancin-Murguzur et al. [28] and Meyer-Jacob et al. [29] applied partial least squares regression to reflectance spectra from dried sediment samples to estimate lake sediment and lake water organic matter concentrations, respectively, in high latitude lakes. Recent developments [30] explore the applicability of this approach to diagnose cyanobacteria-related pigments in lake sediments, which could be useful to reconstruct potentially harmful cyanobacterial blooms in the past.

Whereas the initial paleolimnological work using the VIS-RS methodology focused on the detection of chlorophyll *a* and paleoproductivity reconstructions, Debret et al. [31] expanded the method to include total brightness and first derivative spectra to classify organic-rich sediments, fresh and altered organic matter, iron-rich deposits, carbonate deposits, and clayey deposits. Point measurements were taken on fresh sediment cores with a Minolta CM-2600d Spectrophotometer (360–740 nm spectral range, 10 nm spectral resolution, measurement spot size 5 or 8 mm), an instrument which is often used with multi-sensor core logging equipment (e.g., Geotek Multi-Sensor Core Logger MSCL-S).

Motivated by the need for quantitative reconstructions of climate variables at very high temporal resolutions (annual to subdecadal), von Gunten et al. [10] were the first to apply the approach by Rein and Sirocko (2002) to lake sediments: VIS-RS data were acquired directly from the fresh sediment core using a Spectrolino. In a case study from a lake in central Chile, von Gunten et al. [10] calibrated the VIS-RS index RABD_660;670_ (diagnostic for chlorophylls and productivity) directly to meteorological data and found that, in this particular lake, sedimentary chlorophyll was an excellent predictor for warm season temperature with a remarkably small mean prediction error (RMSEP) of 0.24–0.34 °C. This precise paleo-thermometer was used to establish a quantitative, high-resolution (5 year) temperature reconstruction back to AD 850 [10], which met the high-quality criteria to be included in comprehensive global temperature reconstructions [32,33].

After this proof of concept, calibration and verification methods of quantitative VIS-RS-based climate reconstructions were refined [34] and successfully tested in different parts of the world and types of lake sediments. Using the RABD_660;670_ index, quantitative high-resolution temperature reconstructions were established for lakes in Tasmania [35], Poland [36], and Alaska [37]; a quantitative precipitation reconstruction was also made for a lake in Tasmania [38].

Most recently, it was demonstrated that in maar lakes from the Eifel region of Germany, the RABD_660;670_ index measured by a Spectrolino device could successfully track paleoproductivity back to 60,000 years, showing how rapid temperature changes in the North Atlantic/Greenland domain (i.e., Dansgaard–Oeschger cycles) impacted lake productivity in continental Europe [39].

VIS-RS indices were also developed for quantitative, high-resolution climate reconstructions in clastic sediments. Here, the focus was on spectral index R_570_/R_630_ and related indices that were diagnostic for lithogenic components, mainly illite, chlorite, and mica. In the varved (annually laminated) sediments of Lake Silvaplana (Swiss Alps), Trachsel et al. [40] demonstrated that a combination of VIS-RS indices explained up to 84% of the variance of summer temperatures, and they used this information for a summer temperature reconstruction back to 1150 AD. As in most cases reported here, the length of the reconstruction was limited by the quality of the sediment chronology. The R_570_/R_630_ index was also used for a quantitative 3000-year-long warm season temperature reconstruction from a lake in the central Chilean Andes [41], and a 600-year-long quantitative cold season temperature reconstruction from a proglacial varved lake in Patagonia [42]. In all these examples, the temporal resolution of the climate reconstruction was not limited by the spatial resolution of the Spectrolino measurements, but by the uncertainty of the sediment chronology; this is fundamental for a meaningful climate reconstruction.

The development of VIS-RS techniques and their applications on marine and lake sediments in the past 40 years have shown that, although the instruments were simple compared with state-of-the-art hyperspectral core-scanning devices, VIS-RS techniques with point measurements are suitable for the rapid, inexpensive, and non-destructive biogeochemical analysis of lake sediments. These were the methodological foundations that led to the development of the Specim Single Core Scanner and in situ hyperspectral imaging of lake sediment cores [6].

## 3. Methodological Workflow

### 3.1. Instrument Description and Data Acquisition

**Figure 1:**
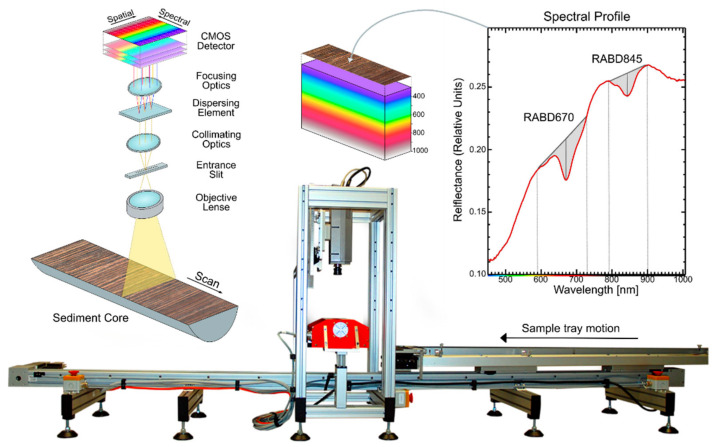
Specim Single Core Scanner showing measurement principle and example of relative absorption band depth indices (RABD_670_ for chloropigments *a* and RABD_845_ for bacteriopheophytin *a*).

The Specim Single Core Scanner system (Spectral Imaging Ltd., Oulu, Finland) is the most commonly used instrument for hyperspectral scanning of lake sediment cores (Figure 1). Hyperspectral images are acquired using a push-broom technique, in which a spectral camera acquires images line-by-line while a motorized sample tray holding a sediment core passes underneath the camera. A domed illumination unit shines indirect light on the sediment sample. All other light sources should be eliminated during data acquisition. Multiple cameras are available, such as the Specim PFD4K-65-V10E for VNIR wavelengths (visible and near infrared; 400–1000 nm) and Specim SWIR camera for longer wavelengths (short-wave infrared, 1000–2500 nm). Technical details of both cameras are summarized in Table 1. Samples up to 12 cm wide and 150 cm long may be analyzed using this system. An interface with a PC enables instrument operation and the storage of raw data files. A dark reference (closed shutter) and white reference (barium sulfate plate) are captured before or after each scan and saved as separate files (more details can be found in the study by Butz et al. [6]).

Prior to scanning, sediment cores are split lengthwise, and the surface is cleaned and flattened to make fine-scale sedimentary features visible and achieve an in-focus image for the entire core. The focus is fixed before scanning and remains constant for a full core segment. Typically, images are acquired of uncovered fresh sediments, but some researchers have used transparent plastic film to reduce the effects of water reflectance [15]. It is also possible to scan dried samples or other materials using this system [6]. Depending on the sediment type, scanning freshly opened cores may yield poor results if sediments are very dark and/or water-saturated. Allowing sediments to dry and oxidize in a cool, dark place for 1–2 days (or more, depending on the sediment type) will often yield higher quality reflectance data. Camera and scanning settings such as exposure and frame rate must be optimized for each core or study site. The field of view and exposure time must be compatible with the speed of the sample tray to ensure undistorted images. Using typical settings and the Specim VNIR camera, a 1-m-long sediment core is scanned in approximately 30 min and produces ~15 GB of raw data.

### 3.2. Data Preprocessing

First, raw reflectance data are normalized to the white and the dark reference, producing a data cube in which spectral data range from 0 to 1 (1 representing 100% reflectance). The following formula is used for normalization [6]:

(1) $${\mathrm{HSI}}_{\mathrm{norm}}=\frac{{\mathrm{HSI}}_{\mathrm{raw}}-{\mathrm{DR}}_{\mathrm{av}}\;}{{\mathrm{WR}}_{\mathrm{av}}-{\mathrm{DR}}_{\mathrm{av}}}\times \frac{{\mathrm{Exp}}_{\mathrm{WR}}}{{\mathrm{Exp}}_{\mathrm{HSI}}}$$

where HSI_raw_ is the raw hyperspectral image, DR_av_ is the dark reference averaged into a single frame, WR_av_ is the white reference averaged into a single frame, Exp_WR_ is the exposure time of the white reference, and Exp_HSI_ is the exposure time of the sample.

After normalization, user-defined subsets of the hyperspectral image are selected to focus on regions of interest (ROIs) for subsequent analyses. Often, two regions of interest are selected: a “big” subset covering the full core face and a “small” subset covering a representative transect downcore. The small subset is used to select the best-preserved portion of the core, avoid irregularities or deformations in the sediment, and reduce computation times compared to using the full image. Optionally, noisy spectral bands can be removed from the data cube during the subset procedure. Masks are used to identify disturbed or unrepresentative areas of the sample, which are removed from subsequent analyses. Additional data processing, such as denoising using filtering techniques, continuum removal, or detrending, may also be applied. When data are acquired using two different sensors (VNIR and SWIR), it is often useful to fuse the two images into a single image prior to post-processing steps. This data fusing requires spatial resolution to be equivalent, so the higher resolution VNIR image is down-sampled to match the lower resolution SWIR image (refer to [15,17] for details).

### 3.3. Data Post-Processing

**Figure 2:**
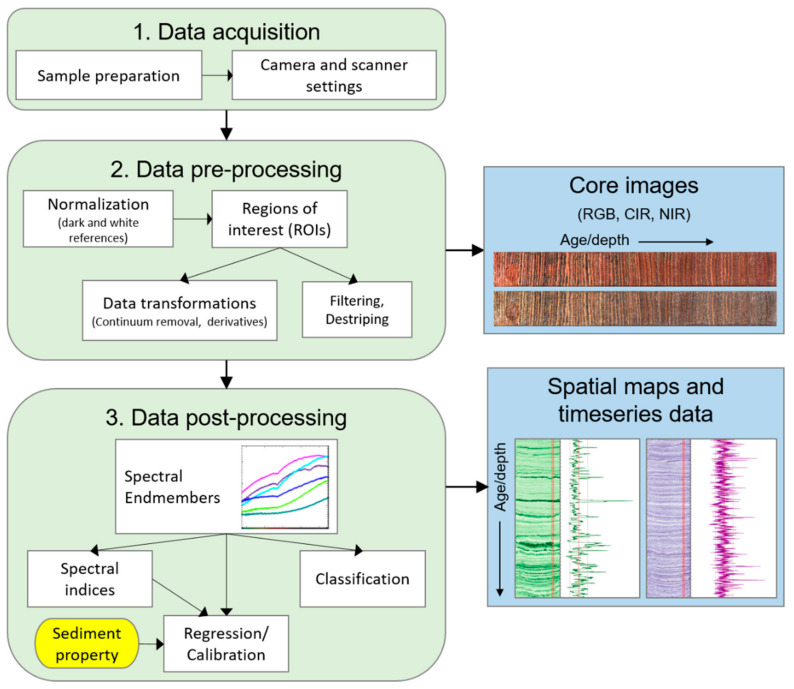
Schematic summary of a typical methodological workflow for hyperspectral imaging analysis of lake sediment cores.

Analysis of spectral data can follow several paths (Figure 2). Images based on RGB (red, green, blue; true color), CIR (color-infrared), or NIR (near-infrared) bands can be generated for the visual inspection of spatial variations. Spectral endmember analysis is utilized to assess the variation of spectral profiles within a dataset and to extract relatively pure components. Endmember spectra can be used to assess notable features (e.g., absorption bands) in the spectra and can be compared with reference spectra from libraries or samples [6]. Subsequent analyses typically fall into one of three categories: spectral indices, classification, and regression.

Spectral indices are relatively simple calculations that can be used to quantify specific features. Indices may be selected based on published literature and/or based on features identified in the spectral endmembers. Commonly used spectral indices include relative absorption band depths (RABD) and relative absorption band areas (RABA) to quantify absorption troughs, and spectral ratios, which measure the slope of the spectral profile between two wavelengths [9]. Table 2 summarizes published spectral indices from HSI studies on lake sediments. These indices are often validated by comparisons with other analyses. For instance, numerous studies have shown that RABD or RABA indices can be calibrated to pigment concentrations (Table 3) [6,12,14,25,43].

Classification refers to a variety of techniques that assign pixels into classes based on the similarity of their spectra. Most often, this is completed using supervised classification, in which target spectra are selected via endmember analysis or by measuring spectra of a known material type (e.g., external samples or regions of interest within the sample) [54]. Then, a dissimilarity measurement, such as spectral angle measure (SAM), is calculated for each pixel to determine how similar its spectrum is to a target spectrum. Finally, each pixel is assigned to one of the target groups, producing a classification map. Additionally, down-core profiles of similarity indices can be used as biogeochemical proxies [55]. Machine learning algorithms have also been shown to accurately classify sediment types based on user-defined reference areas (i.e., regions of interest on the core face that are defined as a particular sediment type) [17]. Unsupervised classification schemes may also be used, meaning no pre-defined target spectra are used. Instead, purely statistical techniques are used to classify regions of an image based on spectral characteristics.

Regression techniques rely on a comparison of HSI data with sediment properties measured using another (often destructive) technique. Careful mapping of the exact sample location is essential for a representative comparison of HSI data with the results of external analyses. Regression models can be applied using the reflectance spectra as input. Alternatively, a combined approach can use spectral indices [13] or similarity indices [55] as inputs to regression models. Numerous studies have calibrated spectral indices to pigment concentrations using regression models, allowing for pigment concentration estimates at the scale of pixels (Table 3). Additionally, predictive models using partial least squares regression and random forest regression with full spectral data as input have been used to predict organic matter concentrations [11] and grain size distributions [15,16] at a high resolution.

## 4. Biogeochemical Interpretation of Sediment Reflectance Spectra

Interpretation of sediment reflectance spectra can be challenging because the spectral profile of a pixel represents a mixture of the spectral signatures of all sedimentary components present in that pixel. Therefore, spectral features (i.e., absorbance troughs) are affected by not only the substance or physical property of interest, but also the sedimentary matrix. Additionally, variations in sedimentary components (from lake to lake but also within one lake) lead to significantly different dominant spectral features. Therefore, calibration and validation with independent measurements using specific analytical techniques (e.g., high-performance liquid chromatography, X-ray diffraction crystallography) are essential for each sediment core before basing key interpretations on spectral features and indices. The interpretation of sediment reflectance profiles should be based on knowledge about the spectral properties of different sediment components, which may be derived from published literature or spectral libraries [56]. Different spectral bands (wavelengths) along the reflectance spectrum are affected by different substances. Spectral features can be associated with single substances or classes of organic or minerogenic components based on absorption related to specific types of chemical bonds [16,57,58,59].

### 4.1. Organic Components

Sediment reflectance data have been used successfully to record high-resolution variations in bulk organic matter as well as specific organic components. Total organic matter content was estimated in sediments from Lake Bourget, France, using reflectance data in the SWIR range input to a partial least squares regression model to predict organic matter concentrations measured in discrete samples [11]. This approach has been widely applied in soil sciences [57,60,61] and can be expected to be applicable to lake sediment. At Lake Linné, Svalbard, spectral endmember analysis enabled the identification of organic material sourced from coal-rich bedrock in the catchment, and a similarity measure (spectral angle) was used to predict organic matter concentrations in the sediments. Van Exem et al. [62] report that charcoal (or altered organic matter) can be recognized based on distinct reflectance patterns identifiable in first derivative spectra plots.

Sedimentary pigments have been a major focus of research using HSI on lake sediment. Often, the most visually dominant feature of sediment spectra is the absorption trough associated with chlorophyll *a* (and degradation products) at around 650–700 nm [9,25]. This absorption feature can be quantified using relative absorption band depth (RABD) or absorption band area (RABA) indices, which have been shown to strongly correlate with high-precision liquid chromatography (HPLC) measurements of chloropigment concentrations in marine [9] and lacustrine sediments [12,14,25,36] (Table 3). Maximal absorbance typically occurs between 660–675 nm, depending on the portions of chlorophyll *a* and *b* and various derivatives (e.g., pheophytin *a*, pheophorbide *a*), as well as the spectral properties of the sediment matrix. RABD_670_ (or similar) has been widely used as an index of green pigments and as a proxy for aquatic productivity in a wide variety of environments, including marine [9,23] and lake sediments [27,36,37,39,63,64], and over timespans greater than 100,000 years [65]. A modified version of the RABD Formula (2) flexibly utilizes the wavelength associated with maximal absorbance, which is preferable for reconstructions of total algal production [14,53,66]:

(2) $${\mathrm{RABD}}_{655-680max}=\left(\frac{X\times R_{590}+\;Y\times R_{730}}{X+Y}\right)/R_{655-680min}$$

where R_λ_ is the reflectance at the wavelength (λ), R_655–680min_ is the trough minimum (i.e., lowest reflectance value measured between 655 and 680 nm), X is the number of spectral bands between R_730_ and the trough minimum, and Y is the number of spectral bands between the trough minimum and R_590_. Endpoint wavelengths (590 and 730 nm in Equation (2)) are selected by examining spectral endmembers to identify the edges of the absorption trough and should be selected from wavelengths that show minimal variability throughout the sample material. Other groups of pigments can also be estimated using the same principle. Bacteriopheophytin *a* (a derivative of bacteriochlorophyll *a* and a biomarker for anoxygenic phototrophic purple sulfur bacteria [67]) absorbs light at approximately 845 nm [6,13,68], and the RABD_845_ index has successfully been calibrated to concentrations of bacteriopheophytin *a* in several studies of lake sediments [6,43,49,50,51,53]. Additionally, an absorption band at 615 nm has been identified as phycocyanin (pigment produced by cyanobacteria [44,69]), and an absorption band at 510 nm has been interpreted as a proxy for carotenoid pigments [9,23]. However, these latter indices have not yet been validated with conventional analytical measurements.

### 4.2. Inorganic Components

Inorganic sedimentary components can also be detected using HSI techniques. Ratios of reflectance R_570_/R_630_ and R_590_/R_690_ have been shown to track variations in lithic content [9,23,40] and are based on decreasing slopes of reflectance across these wavelengths for clay minerals (chlorite, illite, biotite) [9,70,71]. However, if these minerals are not abundant, these ratios will yield noisy or misleading results. The ratio R_850_/R_900_ has been used to track minerogenic input on subantarctic Macquarie Island, where the bedrock geology is dominated by mafic (basaltic) rocks [46]. In calcareous sediments of Lake Jaczno, calcite-rich lamina could be distinguished using a simple threshold applied to total reflectance; this method works because calcite lamina are more reflective than surrounding organic sediments [12]. Hyperspectral images (spectral range of 250–17,000 nm) of dried samples taken from sediment cores from Chew Bahir Basin, Ethiopia provided useful information on minerals (i.e., calcite, smectite, analcime) and sediment geochemistry that compared well with X-ray diffraction mineralogical analysis and X-ray fluorescence elemental analyses [72].

HSI data has been shown to be effective for high-resolution analysis of particle sizes. Jacq et al. [16] used partial least squares regression to predict particle size distributions in sediments of Lake Bourget, France, based on hyperspectral data acquired in the VNIR and SWIR ranges. The resulting high-resolution particle size data enabled the identification of distinct lamina dominated by three different types of sediment: detrital material, small calcite grains, and diatoms or large calcite grains. Ghanbari et al. [15] tested a variety of pre-processing data transformations and predictive models to estimate mean particle size in sediments of six Canadian lakes. A random forest regression model performed best, and it was possible to generate a general model that could be applied to all cores. This model is expected to be applicable in various environments.

## 5. Applications

**Figure 3:**
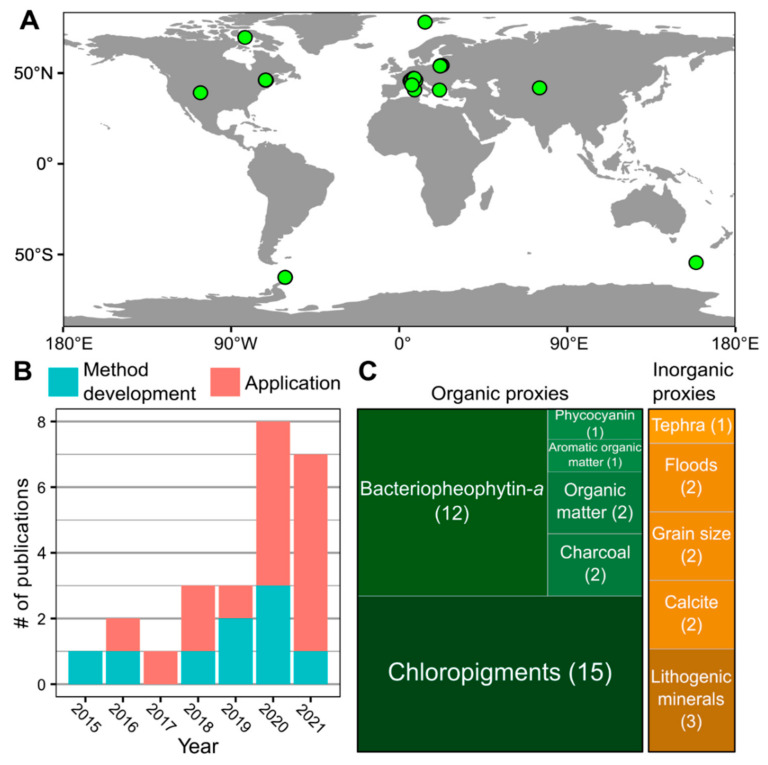
Summary of published hyperspectral imaging datasets from lake sediments in peer-reviewed literature (Table S1). (**A**) Map of study sites with published HSI datasets from lake sediments. (**B**) Number of publications by year showing increasing trend since 2015 (*n* = 25). (**C**) Summary of publications reporting different sedimentary variables from HSI scanning of lake sediments.

Hyperspectral imaging core scanning provides valuable high-resolution biogeochemical data that can help reveal past environmental conditions and processes. Based on a literature search using Google Scholar and Web of Science, we identified 25 published peer-reviewed studies that included original HSI datasets from lake sediment core scanning (Table S1; Figure 3). HSI data are a primary focus in some studies and complementary in others, providing context for the primary analyses. HSI has been applied to lake sediments from across the globe, with the majority of sites being in Europe. The most common application has been the reconstruction of past aquatic productivity based on the absorbance of chloropigments [12,13,14,43,44,45,47,49,50,51,53,66,73,74,75]. Other sedimentary variables interpreted from HSI include bacteriopheophytin *a* [6,12,13,43,44,45,47,49,50,51,53,75], phycocyanin [44], bulk organic matter [11,55], aromatic organic matter [44], charcoal [12,62], tephra [54], flood layers [17,18], particle size [15,16], calcite [12,47], and lithogenic minerals [12,45,46]. In the following sections, we discuss three areas in which HSI has supported key scientific insights.

### 5.1. Seasonal Scale Sedimentation in Varved Lake Sediments

**Figure 4:**
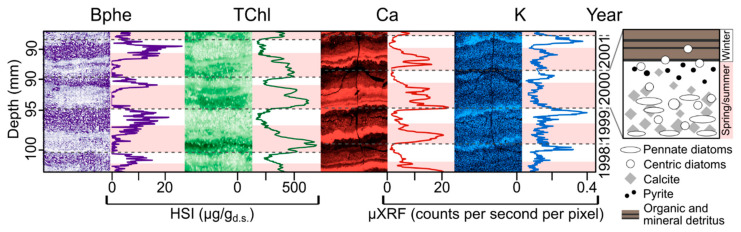
Close-up of varves (representing four years) in sediments of Lake Żabińskie showing seasonal-scale variations in sedimentary pigments (Bphe = bacteriopheophytin *a*, TChl = total chloropigments) inferred from HSI. Bphe indicates the presence of purple sulfur bacteria and strongly anoxic conditions. TChl is a proxy for total algal production. Elemental data from μXRF imaging (Ca, K) are from resin-embedded sediment slabs. Ca represents mainly endogenous calcite precipitation during the warm season. K is indicative of more clastic sediment and typically peaks during ice cover in winter due to the settling of fine minerogenic material and limited primary production/calcite precipitation under ice cover. Gray, dashed lines indicate varve (annual layer) boundaries; pink bars indicate spring/summer layers. Right inset shows schematic microfacies of a single varve year. Modified from Zander et al. [47] and Żarczyński et al. [76].

The extremely high spatial resolution of measurement obtained with HSI makes it possible to investigate seasonal scale sedimentation and biogeochemical processes recorded in varved (annually laminated) sediments. This provides a novel tool to study past seasonal-scale environmental phenomena, such as lake mixing regimes or seasonal climate variability. HSI-inferred sedimentary pigments were used to investigate seasonal-scale varve formation processes in Lake Żabińskie (Figure 4) [47]. Seasonal phytoplankton blooms were recorded by HSI-inferred chloropigments (TChl). Additionally, bacteriopheophytin *a* (Bphe) was used as an indication of lake stratification. Bacteriopheophytin *a* is a biomarker for anoxygenic phototrophic purple sulfur bacteria, which require both light and anoxic/sulfidic conditions to grow [13,67]. Therefore, the presence of this pigment indicates strong stratification of the water column with a shallow chemocline and extensive hypolimnetic anoxia. The HSI pigment data could be linked to micro-X-ray fluorescence (μXRF) imaging data, which was used to identify periods of spring–summer calcite precipitation (Ca) and fine-particle settling in winter under ice cover (K). Zander et al. [47] used these high-resolution imaging techniques to investigate seasonal deposition over the past 54 years, revealing that recent warming temperatures increased the relative amount of biogenic/authigenic sediments and reduced the relative contribution of lithogenic sediment. This research highlights the possibility of high-resolution seasonal–annual scale climate reconstructions based on varved lake sediments using imaging techniques.

Several studies have used the seasonal pattern of bacteriopheophytin *a* to reconstruct past lake mixing regimes. Detectable concentrations of Bphe throughout the varve year indicate the continuous growth of purple sulfur bacteria and are interpreted as being indicative of year-round water-column stratification [43,49,51].

At Lake Bourget, HSI-inferred grain size distributions were used to identify the yearly succession of three distinct endmember sediment components (i.e., large calcite grains/diatoms in late spring, fine calcite grains in summer, and detrital sediments in winter and early spring) [16]. This made it possible to estimate yearly accumulation rates for each of these microfacies. These types of high-resolution analyses of seasonal sedimentation can complement microscope analyses of varves using thin sections by providing rapidly obtained semi-quantitative data at micrometer resolution. Furthermore, such detailed analyses are fundamental for high-resolution paleoenvironmental interpretations.

### 5.2. Long-Term Reconstruction of Aquatic Productivity and Anoxia

**Figure 5:**
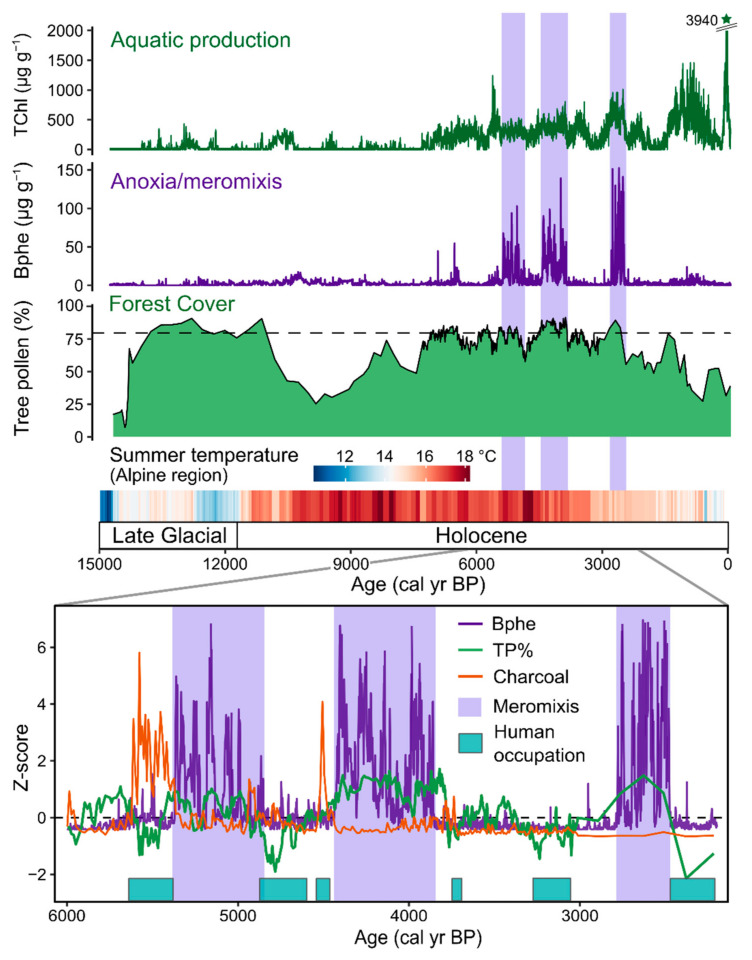
Holocene and Late Glacial (past 15,000 yrs) reconstruction of aquatic productivity and lake mixing from Moossee, Switzerland based on HSI-inferred pigments. Meromixis was inferred from the presence of bacteriopheophytin *a* based on HSI. Meromictic periods (highlighted in purple shading) occurred during periods of closed forest cover (dashed line in the forest cover plot indicates closed forests with 80% tree pollen) and warm summer temperatures. Human-caused forest openings led to periods of greater lake mixing. Modified from Makri et al. [43]. Summer temperature reconstruction inferred for the Alpine region based on chironomids [78].

Several studies have used HSI-inferred sedimentary pigment records for high-resolution reconstructions of aquatic productivity and anoxia over the Holocene and Late Glacial periods (past 20,000 years), providing important context and process understanding relevant for modern cultural eutrophication of lakes. These studies have shown that during warm interstadials (Bölling/Alleröd, 14,500–13,000 years BP) and the Holocene, small, deep lakes in Europe were often strongly stratified and prone to anoxic conditions in environments with closed forests, prior to human disturbance. Forest clearing during the expansion of agriculture in Europe from the Neolithic and Bronze Age onwards led to shifts in lake mixing regimes whereby more intensive lake mixing occurred during periods of forest clearing due to increased wind exposure on these small lakes. The timing of this transition varied depending on the history of deforestation and agricultural development. At Lake Zazari in Greece, lake mixing intensified around 5000 cal year BP during the expansion of Neolithic farming practices [45]. Two sites on the Swiss Plateau (Soppensee [51] and Moossee [43]) showed shifts toward more frequent mixing around 2000–2600 cal year BP (Iron Age, Figure 5). In the Masurian Lakeland of northeast Poland, the human impact on land cover remained weak until approximately the 15th century, and stratified, anoxic conditions persisted until this time at Lake Żabińskie [53] and Lake Łazduny [49]. Sites where high-resolution pollen data are available show that HSI-inferred bacteriopheophytin *a* (Bphe, indicator of anoxic conditions within the photic zone) is typically present during times of closed forest canopy, i.e., >80% tree pollen, showing a close link between (early) human land-use practices and lake biogeochemical conditions (Figure 5) [43,53].

Lake Moossee, Switzerland, provides a clear example of a strong relationship between human land use and lake mixing (Figure 5) [43]. HSI-inferred bacteriopheophytin *a* (Bphe) data, when compared with archaeological information and high-resolution pollen data, show that anoxic and meromictic (meromixis is defined as multi-year periods with no complete lake mixing) periods occurred during times when the lakeshore was not occupied by pile-dwellers and forest coverage was most extensive. The dense forest shielded the small, deep, lake from wind, which led to strong density stratification in the water column and anoxic conditions ideal for Bphe-producing purple sulfur bacteria.

In a more arid environment, Lake Son Kol in Kyrgyzstan provides an example from a large, shallow lake. This lake featured a period of extensive anoxia from about 8500–5200 cal yr BP, driven by warmer temperatures and increased deposition of organic matter, which increased oxygen consumption rates [44].

Reconstructions of aquatic productivity, as recorded by HSI-inferred chloropigments (TChl) in several small temperate lakes, suggest that these lakes experienced gradual increases in productivity during the stable environmental conditions of the Holocene prior to human disturbance [43,49,50,51,53] (Figure 5). This gradual long-term increase in productivity can be attributed to the natural development of nutrient pools in lakes and their catchments [77]. During the past 100–200 years, intensive agriculture and grazing led to drastic increases in aquatic productivity (cultural eutrophication) due to increased nutrient delivery from soil erosion and fertilizer use [43,49,53].

**Figure 6:**
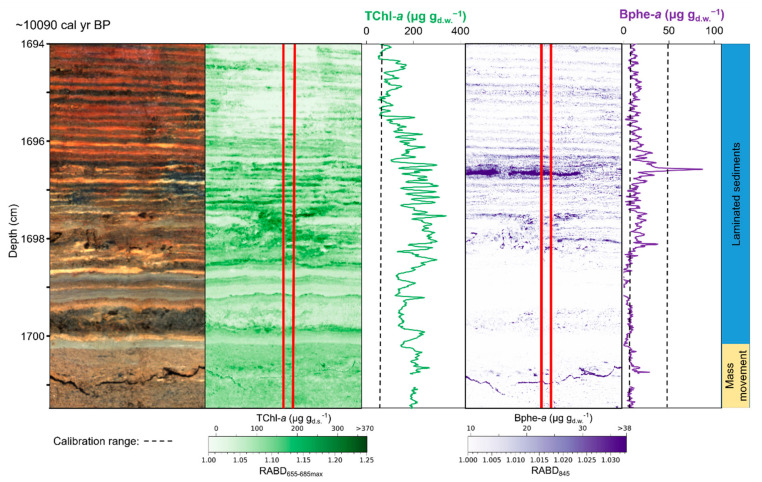
Example of phototrophic community response to mass movement event from Lake Żabińskie, Poland. Left to right: RGB image of sediment section, map and downcore profile of chloropigments *a* (RABD_655–685max_), map and downcore profile of bacteriopheophytin *a* (RABD_845_). The upper boundary of a mass movement deposit is located just below 1700 cm (sediment core depth), with calcareous biochemical varves overlying the event deposit. Phytoplankton production (TChl *a*) recovered immediately after the mass movement event, whereas purple sulfur bacteria (Bphe *a*) production was delayed by 2–5 years. Modified from Zander et al. [53].

The high spatial (temporal) resolution of HSI data, particularly from varved sediments, provides unique information about rapid environmental changes and short-lived events. For instance, mass movements, such as slumps and turbidity currents, are known to disturb stratification within lakes. At Lake Żabińskie, the reestablishment of lake stratification following a mass movement event could be constrained to within 2–5 years following the event (Figure 6) [53]. Also in Lake Żabińskie, reduced lake mixing in response to reforestation around 600 CE led to a ten-fold increase in Bphe concentrations within 2 years, demonstrating a clear threshold response to an ecological change [53]. With HSI it is possible to pinpoint changes at a sub-mm scale, and with varves, to determine precisely the rate of change.

### 5.3. Identification of Stratigraphic Changes and Sedimentary Structures

**Figure 7:**
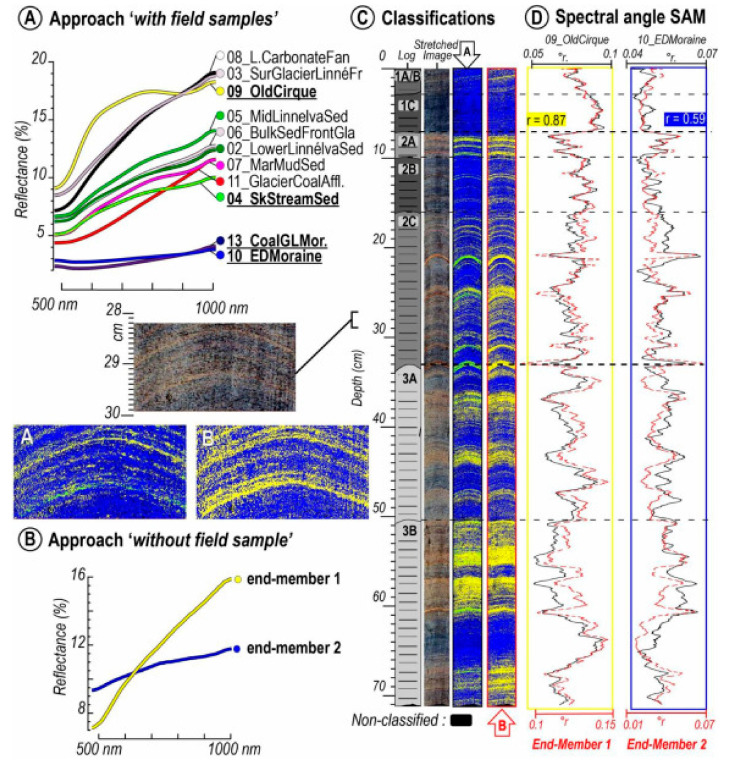
Example of hyperspectral image classification techniques to distinguish sedimentary sources at Lake Linné, Svalbard [55]. (**A**) Reflectance spectra of rock and sediment samples obtained from the catchment area. (**B**) Endmember spectra identified in the sediment core. (**C**) Core image, and classification maps based on similarity to spectra in (**A**,**B**,**D**) Downcore profiles of similarity scores (spectral angle) for endmembers 1 and 2, as well as the field samples most similar to those two endmembers. Endmember 1 is composed of fine-grained clastic sediments, whereas Endmember 2 represents sediments with greater organic matter content, and is similar to field samples taken from coal-rich deposits in the catchment area (13_CoalGLMor and 10_EDMoraine). Reprinted from Van Exem et al. [55] with permission from Elsevier.

Sediment stratigraphy represents the classification of sediment layers based on their properties and is fundamental for paleoenvironmental research because changes in sediment properties are driven by changing environmental conditions. Hyperspectral imaging of lake sediments has proven useful for discriminating stratigraphic changes at scales ranging from micrometers to meters. A common approach is to identify reference areas on a core image that represent a certain sediment type (lithotype). Then, an image classification algorithm is used to identify other areas in the sediment core with similar properties. This approach was used to discriminate layers deposited during flood events from continuous background sedimentation at Lake Bourget, France [18]. Recently, Jacq et al. [17] expanded this work to three lakes in France using several machine learning algorithms to successfully identify flood event layers. These studies demonstrate great promise for developing records of flood frequency prior to historical documentation or instrumental monitoring. Long-term records are necessary for robust estimates of extreme flood event probabilities [79]. Butz et al. [6] used image classification to identify clay-rich and charcoal-rich layers associated with erosional events and fires, respectively, in sediments of Lake Jaczno (Poland). Tephra (volcanic ash) deposits could also be distinguished from background sediments in three Antarctic lakes, including some tephra layers that were not initially detected by eye [40]. Tephra deposits are important stratigraphic markers used to correlate geological records from diverse sites, including lake and marine sediments, ice cores, and peat bogs [80]. In an effort to characterize sources of sediments in an arctic lake, the sediments of Lake Linné, Svalbard, were classified based on the similarity of their reflectance spectra to samples of source material from the catchment (Figure 7) [55]. This enabled the authors to identify organic-rich layers in the sediment core that were derived from coal-rich sedimentary rocks in the catchment area. It was also revealed that glacial erosion of the coal-bearing bedrock units increased the delivery of organic material to the lake. This study provides a promising example of a new approach to source-to-sink sedimentological studies at the lamina scale.

Biogeochemical data derived from HSI methods have also been used in conjunction with other high-resolution datasets (i.e., μXRF, magnetic susceptibility, gamma-ray density) to identify stratigraphic changes in sediment cores [43,49,53]. This work is often performed by utilizing multivariate clustering algorithms. Stratigraphically constrained cluster analysis [81] on such datasets provides an objective method for identifying stratigraphic units that can be compared with conventional sediment descriptions [43,51,53]. Alternatively, unconstrained cluster analysis provides information about sediment type variations (lithotypes or facies) at high-resolution [43,49,50,53]. These approaches complement conventional sedimentological tools and provide automated high-resolution assessments of sedimentological variations for long sedimentary records [82].

## 6. Potential Uses and Limitations

HSI offers several clear advantages in comparison with conventional analytical techniques that require time-consuming and destructive laboratory analysis. HSI provides data at an extremely high spatial resolution (40–200 μm) with relatively minimal time, labor, and cost. Additionally, spatial variations in sedimentary characteristics captured by HSI can provide important information about sedimentation and diagenetic processes, as well as the representativeness of a sample area. Sediments that feature substantial variability at μm-to-cm scales are particularly well suited for HSI analysis because conventional methods are unable to capture such high-resolution spatial (and temporal) variability. Varved sediments preserve annual variations in sediment composition and are particularly well suited for HSI. Considering that lake sediments typically accumulate at rates of approximately 0.1 to 10 mm per year, the 0.04–0.2 mm resolution of HSI data is ideal for studying seasonal-to-annual-scale variability. Nevertheless, HSI is efficient for investigating long records covering tens of meters and thousands of years (or greater). HSI is most valuable for studies that require high-resolution data, including studies on high-frequency climate oscillations (e.g., El Niño–Southern Oscillation), thresholds and tipping points, and short-lived events (e.g., floods, fires, heat waves). HSI analysis is most effective when complemented by traditional sedimentological analysis, X-ray florescence core scanning, HPLC analysis, or other methods [83]. These techniques can provide validation or calibration of hyperspectral-based data, as well as complementary data that may aid interpretations. Micro-X-ray fluorescence (μXRF) imaging can be combined with HSI for extremely detailed investigations of sedimentary composition at a μm scale. For example, the combination of these techniques provided insights on how seasonal weather conditions affected varve composition [47].

Biogeochemical analysis of sediments using HSI is limited by inherent uncertainty related to the interpretation of reflectance spectra. Similar spectral features can be produced by multiple substances or mixtures of substances. Therefore, HSI data should be validated using independent information. The use of established indices in well-characterized sediments may be possible without independent validation. However, significant questions remain about calibration and predictive models based on HSI data. For example, the RABD_670_ index has been applied in a wide variety of sites and, therefore, is expected to successfully track changing aquatic production at most sites [27]. However, no universal calibration model has been developed for relating RABD_670_ to chloropigment concentrations. Table 3 summarizes pigment calibration models obtained from different HSI studies. The variation of calibration slopes could reflect methodological choices (including HSI data acquisition and processing, or pigment analysis methods) and/or variable sedimentary matrices. Although the majority of calibration slopes are similar, especially for studies using the same pigment measurement method, some studies demonstrate distinctly different slopes, suggesting that site-to-site variations may lead to a distinctly different relationship between RABD index values and pigment concentrations.

HSI analysis of sediments can be limited or affected by the characteristics of lake sediments. Water-saturated sediments can cause interference through spectral scattering, and water causes prominent absorption bands in the SWIR range that interfere with the reflectance signal of the sediments. Drying sediments may be useful in some cases, though this typically leads to cracking/deformation of intact sediment cores. Degradation and oxidation of sediments during core opening and storage should also be considered. These effects may lead to the loss of organic components or variations in the reflectance data due to oxidation during storage. Certain sediments may be too dark to obtain reliable data (i.e., near total light absorption). Low reflectance can lead to biases in calibration models applied to spectral indices for pigments [52]. The problem of near total light absorption has, so far, prevented the use of resin-embedded sediment slabs for HSI scanning despite the much better preservation of fine sediment structures in resin-embedded slabs [84].

Research on HSI applied to lake sediments is still in its initial development, and much potential remains for advancement in data processing techniques and the interpretation of large HSI datasets. Continued research can be expected to include new applications, better interpretation models of sediment reflectance, and advanced numerical tools applied to HSI datasets.

## 7. Conclusions and Outlook

In this review, we summarized the recent developments of in situ hyperspectral imaging applied to lake sediment cores for biogeochemical analysis. This promising field of research is growing rapidly since the first published demonstration of the technique in 2014 [85]. HSI offers important advantages over conventional biogeochemical methods: (1) HSI is non-destructive, meaning no sediment is consumed by HSI scanning and the sediment core can be used for further subsequent analyses; (2) data can be acquired at an extremely high resolution (40–300 μm), and (3) data can be acquired rapidly and at low cost. HSI can be used to assess a wide variety of sedimentary characteristics and components including tephra, grain size, flood deposits, organic matter, and pigments. The interpretation of sediment reflectance data typically requires validation or expert knowledge about the sediment composition. Ongoing research will refine and expand the set of numerical tools used to translate hyperspectral images into quantitative or semi-quantitative biogeochemical datasets and paleoenvironmental interpretations.

To date, the majority of paleoenvironmental reconstructions based on HSI data have utilized spectral indices that quantify pigment groups (e.g., chloropigments, bacteriopheophytin). Such indices can be calibrated to concentrations measured on discrete samples using conventional wet-chemical techniques. HSI-inferred pigment records have been used to track changes in lake productivity, organic matter sources, and lake mixing over thousands of years, leading to important insights about relationships between climate, land cover, and lake ecosystems. These studies provide examples of the potential of HSI for paleoenvironmental research, which can only be expected to grow in the future.

Several areas of opportunity regarding HSI applied to lake sediments should be considered in future research. One important task is to assess to what extent methods can be universally applied to different sites with variable environments and sedimentary compositions. For instance, can universal calibration models be established to convert HSI data to sedimentary properties of interest? This likely requires better standardization of methods between various research groups as this field of study continues to grow. Expanding the geographic scope of HSI investigations of sediment across the globe is an important goal. Currently, the vast majority of HSI studies on lake sediment have been from lakes in Europe. Additionally, HSI should be applied to records covering longer time periods. The longest published HSI record to date is 20,000 years [45]. Applying HSI to records covering multiple glacial–interglacial cycles could yield valuable information about high-frequency climate variability, but may also present additional challenges related to diagenetic processes such as the degradation and diagenesis of pigments. Technological advances can be expected to improve HSI data acquisition. Currently, low signal-to-noise ratios in the lower wavelengths (<450 nm; [6]) present a challenge for reliably detecting substances that absorb in this range, such as carotenoid pigments. Future generations of hyperspectral cameras will likely feature improved signal-to-noise ratios and greater spectral resolution. Higher spectral resolution could enable the use of spectral deconvolution methods to HSI datasets from lake sediments, similar to those used for the analysis of pigments in solvents [86,87]. Innovative and advanced numerical techniques, including machine learning algorithms, will improve the efficiency and reliability of the HSI analysis of lake sediments. Advanced numerical approaches also have the potential for new insights on and interpretations of sediment reflectance data.

## Figures and Tables

**Table 1:** Summary of technical specifications for hyperspectral cameras available for sediment core scanning (Spectral Imaging Ltd., Oulu, Finland).

Parameter	Specim PFD4K-65-V10E	Specim Spectral Camera SWIR
Spectral range	400–1000 nm	1000–2500 nm
Spectral sampling resolution	0.78–6.27 nm	5.6 nm
Spatial resolution (pixel size)	40–90 µm	130–310 μm
Field of view width	50–120 mm	50–120 mm
Radiometric resolution (Bit)	12	16

**Table 2:** List of spectral indices used in HSI studies of lake sediments.

Index (Alternatives)	Proxy	Example Locations	Example References
RABD_670_^1^ (RABA_650–700_; RABD_655–685max;_ RABD_670_/Rmean, RABA_630–700_/R_670_)	Chloropigments *a* (aquatic production)	Lake Jaczno, Poland; Lake Lugano (Ponte Tresa basin), Switzerland	Butz et al., 2017 [12]; Schneider et al., 2018 [14]
RABD_845_ (RABA_750–900_/R_845_)	Bacteriopheophytin *a* (water column anoxia)	Lake Jaczno, Poland; Lake Moossee, Switzerland	Butz et al., 2016 [13]; Makri et al., 2020 [43]
RABD_615_ (RABA_600–630_/R_615_)	Phycoyanin (cyanobacteria)	Lake Son Kol, Kyrgyzstan	Sorrel et al., 2021 [44];
RABA_1660–1690_/R_1670_	Aromatic organic matter (terrestrial organic matter)	Lake Son Kol, Kyrgyzstan	Sorrel et al., 2021 [44]
R_570_/R_630_^1^ (R_590_/R_690)_	Lithogenic material (chlorite, illite, biotite)	Lake Zazari, Greece	Gassner et al., 2020 [45]
R_850_/R_900_	Lithogenic material (Basaltic lithics)	Emerald Lake, Australia	Saunders et al., 2018 [46]
R_mean_	Unspecific; calcite	Lake Jaczno, Poland; Lake Żabińskie, Poland	Butz et al., 2017 [12]; Zander et al., 2021 [47]

^1^ Original reference: Rein and Sirocko, 2002 [9].

**Table 3:** Summary of published pigment calibrations using hyperspectral imaging spectral indices.

		RMSE (μg/g)		R-sq		Slope (μg/Index)		Pigment Method
Publication	Lake	Bphe *a*	TChl *a*	Bphe *a*	TChl *a*	Bphe *a*	TChl *a*	
Butz et al., 2015 [6]	Jaczno, Poland	3.0	_	0.89	_	644	_	HPLC
Butz et al., 2017 [12]	Jaczno, Poland	_	36.8/ 20.1	_	0.74/ 0.96	_	2355/1428	Spectrophoto-meter
Schneider et al., 2018 [14]	Lugano (Ponte Tresa basin), Switzerland	_	123.2	_	0.83	_	454	HPLC
Wienhues, 2019 [48]	Rzęśniki, Poland	18.8	26.8	0.87	0.78	1867	1118	HPLC
Sanchini et al., 2020 [49]	Łazduny, Poland	24.0	103.5	0.89	0.89	761	2132	Spectrophoto-meter
Makri et al., 2020 [43]	Moossee, Switzerland	3.2	188.7	0.92	0.87	964	6949	Spectrophoto-meter
Makri et al., 2021 [50]	Jaczno, Poland	3.1	22.0	0.95	0.91	680	1558	Spectrophoto-meter
Tu et al., 2021 [51]	Soppensee, Switzerland	15.9	40.4	0.96	0.84	787	1538	Spectrophoto-meter
Hächler, 2021 [52]	Mezzano, Italy	3.4	47.3	0.69	0.79	509	1528	Spectrophoto-meter
Zander et al., 2021 [53]	Żabińskie, Poland	5.7	77.1	0.80	0.93	861	1558	Spectrophoto-meter

## Data Availability

This study reports no new datasets.

## Supplementary Materials

The following are available online at https://www.mdpi.com/article/10.3390/jimaging8030058/s1, Table S1: List of peer-reviewed articles presenting original HSI datasets from in situ core scanning of lake sediments.

## References

[1] Smol J.P., Birks H.J.B., Last W.M. (2001). Tracking Environmental Change Using Lake Sediments. Volume 3: Terrestrial, Algal, and Siliceous Indicators.

[2] Last W.M., Smol J.P. (2001). Tracking Environmental Change Using Lake Sediments. Volume 2: Physical and Geochemical Methods.

[3] Rothwell R.G., Rack F.R. (2006). New techniques in sediment core analysis: An introduction. Geol. Soc. Lond. Spéc. Publ..

[4] Francus P. (2005). Image Analysis, Sediments and Paleoenvironments.

[5] Croudace I., Rothwell R. (2015). Micro-XRF Studies of Sediment Cores: Applications of a Non-Destructive Tool for the Environmental Sciences.

[6] Butz C., Grosjean M., Fischer D., Wunderle S., Tylmann W., Rein B. (2015). Hyperspectral imaging spectroscopy: A promising method for the biogeochemical analysis of lake sediments. J. Appl. Remote Sens..

[7] Hajdas I., Ascough P., Garnett M.H., Fallon S.J., Pearson C.L., Quarta G., Spalding K.L., Yamaguchi H., Yoneda M. (2021). Radiocarbon dating. Nat. Rev. Methods Prim..

[8] Zolitschka B., Francus P., Ojala A.E., Schimmelmann A. (2015). Varves in lake sediments—A review. Quat. Sci. Rev..

[9] Rein B., Sirocko F. (2002). In-situ reflectance spectroscopy—Analysing techniques for high-resolution pigment logging in sediment cores. Geol. Rundsch..

[10] Von Gunten L., Grosjean M., Rein B., Urrutia R., Appleby P. (2009). A quantitative high-resolution summer temperature reconstruction based on sedimentary pigments from Laguna Aculeo, central Chile, back to AD 850. Holocene.

[11] Jacq K., Perrette Y., Fanget B., Sabatier P., Coquin D., Martinez-Lamas R., Debret M., Arnaud F. (2019). High-resolution prediction of organic matter concentration with hyperspectral imaging on a sediment core. Sci. Total Environ..

[12] Butz C., Grosjean M., Goslar T., Tylmann W. (2017). Hyperspectral imaging of sedimentary bacterial pigments: A 1700-year history of meromixis from varved Lake Jaczno, northeast Poland. J. Paleolimnol..

[13] Butz C., Grosjean M., Poraj-Górska A., Enters D., Tylmann W. (2016). Sedimentary Bacteriopheophytin a as an indicator of meromixis in varved lake sediments of Lake Jaczno, north-east Poland, CE 1891–2010. Glob. Planet. Chang..

[14] Schneider T., Rimer D., Butz C., Grosjean M. (2018). A high-resolution pigment and productivity record from the varved Ponte Tresa basin (Lake Lugano, Switzerland) since 1919: Insight from an approach that combines hyperspectral imaging and high-performance liquid chromatography. J. Paleolimnol..

[15] Ghanbari H., Jacques O., Adaïmé M.-É., Gregory-Eaves I., Antoniades D. (2020). Remote Sensing of Lake Sediment Core Particle Size Using Hyperspectral Image Analysis. Remote Sens..

[16] Jacq K., Giguet-Covex C., Sabatier P., Perrette Y., Fanget B., Coquin D., Debret M., Arnaud F. (2019). High-resolution grain size distribution of sediment core with hyperspectral imaging. Sediment. Geol..

[17] Jacq K., Rapuc W., Benoit A., Coquin D., Fanget B., Perrette Y., Sabatier P., Wilhelm B., Debret M., Arnaud F. (2021). Sedimentary structure discrimination with hyperspectral imaging in sediment cores. Sci. Total Environ..

[18] Rapuc W., Jacq K., Develle-Vincent A.-L., Sabatier P., Fanget B., Perrette Y., Coquin D., Debret M., Wilhelm B., Arnaud F. (2020). XRF and hyperspectral analyses as an automatic way to detect flood events in sediment cores. Sediment. Geol..

[19] Swain E.B. (1985). Measurement and interpretation of sedimentary pigments. Freshw. Biol..

[20] Züllig H., Rheineck S.G. (1985). Pigmente phototropher Bakterien in Seesedimenten und ihre Bedeutung für die Seenforschung-Mit Ergebnissen aus dem Lago Cadagno, Rotsee und Lobsigensee. Swiss J. Hydrol..

[21] Deaton B.C., Balsam W.L. (1991). Visible spectroscopy; a rapid method for determining hematite and goethite concentration in geological materials. J. Sediment. Res..

[22] Balsam W.L., Deaton B.C. (1996). Determining the composition of late Quaternary marine sediments from NUV, VIS, and NIR diffuse reflectance spectra. Mar. Geol..

[23] Rein B., Lückge A., Reinhardt L., Sirocko F., Wolf A., Dullo W.-C. (2005). El Niño variability off Peru during the last 20,000 years. Paleoceanography.

[24] Das B., Vinebrooke R.D., Sanchez-Azofeifa A., Rivard B., Wolfe A.P. (2005). Inferring sedimentary chlorophyll concentrations with reflectance spectroscopy: A novel approach to reconstructing historical changes in the trophic status of mountain lakes. Can. J. Fish. Aquat. Sci..

[25] Wolfe A.P., Vinebrooke R.D., Michelutti N., Rivard B., Das B. (2006). Experimental calibration of lake-sediment spectral reflectance to chlorophyll a concentrations: Methodology and paleolimnological validation. J. Paleolimnol..

[26] Michelutti N., Blais J.M., Cumming B.F., Paterson A.M., Rühland K., Wolfe A.P., Smol J.P. (2010). Do spectrally inferred determinations of chlorophyll a reflect trends in lake trophic status?. J. Paleolimnol..

[27] Michelutti N., Smol J. (2016). Visible spectroscopy reliably tracks trends in paleo-production. J. Paleolimnol..

[28] Ancin-Murguzur F.J., Brown A.G., Clarke C., Sjøgren P., Svendsen J.I., Alsos I.G. (2020). How well can near infrared reflectance spectroscopy (NIRS) measure sediment organic matter in multiple lakes?. J. Paleolimnol..

[29] Meyer-Jacob C., Michelutti N., Paterson A.M., Monteith D., Yang H., Weckström J., Smol J.P., Bindler R. (2017). Inferring Past Trends in Lake Water Organic Carbon Concentrations in Northern Lakes Using Sediment Spectroscopy. Environ. Sci. Technol..

[30] Favot E.J., Hadley K.R., Paterson A.M., Michelutti N., Watson S.B., Zastepa A., Hutchinson N.J., Vinebrooke R.D., Smol J.P. (2020). Using visible near-infrared reflectance spectroscopy (VNIRS) of lake sediments to estimate historical changes in cyanobacterial production: Potential and challenges. J. Paleolimnol..

[31] Debret M., Sebag D., Desmet M., Balsam W., Copard Y., Mourier B., Susperrigui A.-S., Arnaud F., Bentaleb I., Chapron E. (2011). Spectrocolorimetric interpretation of sedimentary dynamics: The new “Q7/4 diagram”. Earth Sci. Rev..

[32] Emile-Geay J., McKay N.P., Kaufman D.S., von Gunten L., Wang J., Anchukaitis K., Abram N., Addison J., Curran M.A., PAGES2k Consortium (2017). A global multiproxy database for temperature reconstructions of the Common Era. Sci. Data.

[33] Ahmed M., Anchukaitis K.J., Asrat A., Borgaonkar H.P., Braida M., Buckley B.M., Büntgen U., Chase B.M., Christie D.A., Cook E.R. (2013). Consortium Continental-scale temperature variability during the past two millennia. Nat. Geosci..

[34] Von Gunten L., Grosjean M., Kamenik C., Fujak M., Urrutia R. (2012). Calibrating biogeochemical and physical climate proxies from non-varved lake sediments with meteorological data: Methods and case studies. J. Paleolimnol..

[35] Saunders K., Grosjean M., Hodgson D. (2013). A 950 yr temperature reconstruction from Duckhole Lake, southern Tasmania, Australia. Holocene.

[36] Amann B., Lobsiger S., Fischer D., Tylmann W., Bonk A., Filipiak J., Grosjean M. (2014). Spring temperature variability and eutrophication history inferred from sedimentary pigments in the varved sediments of Lake Żabińskie, north-eastern Poland, AD 1907–2008. Glob. Planet. Chang..

[37] Boldt B.R., Kaufman D.S., McKay N.P., Briner J.P. (2015). Holocene summer temperature reconstruction from sedimentary chlorophyll content, with treatment of age uncertainties, Kurupa Lake, Arctic Alaska. Holocene.

[38] Saunders K., Kamenik C., Hodgson D., Hunziker S., Siffert L., Fischer D., Fujak M., Gibson J., Grosjean M. (2012). Late Holocene changes in precipitation in northwest Tasmania and their potential links to shifts in the Southern Hemisphere westerly winds. Glob. Planet. Chang..

[39] Sirocko F., Martínez-García A., Mudelsee M., Albert J., Britzius S., Christl M., Diehl D., Diensberg B., Friedrich R., Fuhrmann F. (2021). Muted multidecadal climate variability in central Europe during cold stadial periods. Nat. Geosci..

[40] Trachsel M., Grosjean M., Schnyder D., Kamenik C., Rein B. (2010). Scanning reflectance spectroscopy (380–730 nm): A novel method for quantitative high-resolution climate reconstructions from minerogenic lake sediments. J. Paleolimnol..

[41] De Jong R., Von Gunten L., Maldonado A., Grosjean M. (2013). Late Holocene summer temperatures in the central Andes reconstructed from the sediments of high-elevation Laguna Chepical, Chile (32 S). Clim. Past.

[42] Elbert J., Jacques-Coper M., Van Daele M., Urrutia R., Grosjean M. (2015). A 600 years warm-season temperature record from varved sediments of Lago Plomo, Northern Patagonia, Chile (47 S). Quat. Int..

[43] Makri S., Rey F., Gobet E., Gilli A., Tinner W., Grosjean M. (2020). Early human impact in a 15,000-year high-resolution hyperspectral imaging record of paleoproduction and anoxia from a varved lake in Switzerland. Quat. Sci. Rev..

[44] Sorrel P., Jacq K., Van Exem A., Escarguel G., Dietre B., Debret M., McGowan S., Ducept J., Gauthier E., Oberhänsli H. (2020). Evidence for centennial-scale Mid-Holocene episodes of hypolimnetic anoxia in a high-altitude lake system from central Tian Shan (Kyrgyzstan). Quat. Sci. Rev..

[45] Gassner S., Gobet E., Schwörer C., Van Leeuwen J., Vogel H., Giagkoulis T., Makri S., Grosjean M., Panajiotidis S., Hafner A. (2019). 20,000 years of interactions between climate, vegetation and land use in Northern Greece. Veg. Hist. Archaeobotany.

[46] Saunders K.M., Roberts S., Perren B., Butz C., Sime L., Davies S., Van Nieuwenhuyze W., Grosjean M., Hodgson D.A. (2018). Holocene dynamics of the Southern Hemisphere westerly winds and possible links to CO_2_ outgassing. Nat. Geosci..

[47] Zander P.D., Żarczyński M., Tylmann W., Rainford S.-K., Grosjean M. (2021). Seasonal climate signals preserved in biochemical varves: Insights from novel high-resolution sediment scanning techniques. Clim. Past.

[48] Wienhues G. (2019). Multi-Proxy Reconstruction of Holocene Environmental Change from Sediments of Lake Rzęśniki, Northeast Poland. Master’s Thesis.

[49] Sanchini A., Szidat S., Tylmann W., Vogel H., Wacnik A., Grosjean M. (2020). A Holocene high-resolution record of aquatic productivity, seasonal anoxia and meromixis from varved sediments of Lake Łazduny, North-Eastern Poland: Insight from a novel multi-proxy approach. J. Quat. Sci..

[50] Makri S., Lami A., Tu L., Tylmann W., Vogel H., Grosjean M. (2021). Holocene phototrophic community and anoxia dynamics in meromictic Lake Jaczno (NE Poland) using high-resolution hyperspectral imaging and HPLC data. Biogeosciences.

[51] Tu L., Gilli A., Lotter A.F., Vogel H., Moyle M., Boyle J.F., Grosjean M. (2021). The nexus among long-term changes in lake primary productivity, deep-water anoxia, and internal phosphorus loading, explored through analysis of a 15,000-year varved sediment record. Glob. Planet. Chang..

[52] Hächler L. (2021). High-Resolution Record of Primary Productivity and Anoxia in the Context of the Environmental History of Lago di Mezzano. Master’s Thesis.

[53] Zander P.D., Żarczyński M., Vogel H., Tylmann W., Wacnik A., Sanchini A., Grosjean M. (2021). A high-resolution record of Holocene primary productivity and water-column mixing from the varved sediments of Lake Żabińskie, Poland. Sci. Total Environ..

[54] Aymerich I.F., Oliva M., Giralt S., Martín-Herrero J. (2016). Detection of Tephra Layers in Antarctic Sediment Cores with Hyperspectral Imaging. PLoS ONE.

[55] Van Exem A., Debret M., Copard Y., Verpoorter C., De Wet G., Lecoq N., Sorrel P., Werner A., Roof S., Laignel B. (2019). New source-to-sink approach in an arctic catchment based on hyperspectral core-logging (Lake Linné, Svalbard). Quat. Sci. Rev..

[56] Kokaly R.F., Clark R.N., Swayze G.A., Livo K.E., Hoefen T.M., Pearson N.C., Wise R.A., Benzel W.M., Lowers H.A., Driscoll R.L. (2017). USGS Spectral Library Version 7 Data: US Geological Survey Data Series 1035.

[57] Rossel R.V., Behrens T. (2010). Using data mining to model and interpret soil diffuse reflectance spectra. Geoderma.

[58] Bishop J., Lane M.D., Dyar M.D., Brown A.J. (2008). Reflectance and emission spectroscopy study of four groups of phyllosilicates: Smectites, kaolinite-serpentines, chlorites and micas. Clay Miner..

[59] Clark R.N., King T.V.V., Klejwa M., Swayze G.A., Vergo N. (1990). High spectral resolution reflectance spectroscopy of minerals. J. Geophys. Res. Solid Earth.

[60] Viscarra Rossel R.A., Behrens T., Ben-Dor E., Brown D.J., Demattê J.A.M., Shepherd K.D., Shi Z., Stenberg B., Stevens A., Adamchuk V. (2016). A global spectral library to characterize the world’s soil. Earth Sci. Rev..

[61] Peng X., Shi T., Song A., Chen Y., Gao W. (2014). Estimating Soil Organic Carbon Using VIS/NIR Spectroscopy with SVMR and SPA Methods. Remote Sens..

[62] Van Exem A., Debret M., Copard Y., Vannière B., Sabatier P., Marcotte S., Laignel B., Reyss J.-L., Desmet M. (2018). Hyperspectral core logging for fire reconstruction studies. J. Paleolimnol..

[63] Meyer I., Van Daele M., Fiers G., Verleyen E., De Batist M., Verschuren D. (2017). Sediment reflectance spectroscopy as a paleo-hydrological proxy in East Africa. Limnol. Oceanogr. Methods.

[64] Chen Q., Liu X., Nie Y., Sun L. (2013). Using visible reflectance spectroscopy to reconstruct historical changes in chlorophyllaconcentration in East Antarctic ponds. Polar Res..

[65] Axford Y., Briner J.P., Cooke C.A., Francis D.R., Michelutti N., Miller G.H., Smol J.P., Thomas E.K., Wilson C.R., Wolfe A.P. (2009). Recent changes in a remote Arctic lake are unique within the past 200,000 years. Proc. Natl. Acad. Sci. USA.

[66] Tu L., Zander P., Szidat S., Lloren R., Grosjean M. (2020). The influences of historic lake trophy and mixing regime changes on long-term phosphorus fraction retention in sediments of deep eutrophic lakes: A case study from Lake Burgäschi, Switzerland. Biogeosciences.

[67] Damsté J.S.S., Schouten S. (2006). Biological markers for anoxia in the photic zone of the water column. Handbook of Environmental Chemistry, Volume 2: Reactions and Processes.

[68] Ji J., Balsam W., Shen J., Wang M., Wang H., Chen J. (2009). Centennial blooming of anoxygenic phototrophic bacteria in Qinghai Lake linked to solar and monsoon activities during the last 18,000 years. Quat. Sci. Rev..

[69] Yackulic E. (2017). Productivity and Temperature Variability Over the Past 15000 Years at a Small Alpine Lake in the Southern San Juan Mountains, Colorado. Master’s Thesis.

[70] USGS Digital Spectral Library, splib06a. http://speclab.cr.usgs.gov/spectral-lib.html.

[71] Rein B., Lückge A., Sirocko F. (2004). A major Holocene ENSO anomaly during the Medieval period. Geophys. Res. Lett..

[72] Arnold G.E., Foerster V., Trauth M.H., Lamb H., Schaebitz F., Asrat A., Szczech C., Günter C. (2021). Advanced Hyperspectral Analysis of Sediment Core Samples from the Chew Bahir Basin, Ethiopian Rift, in the Spectral Range from 0.25 to 17 µm: Support for Climate Proxy Interpretation. Front. Earth Sci..

[73] Chiaia-Hernández A.C., Zander P.D., Schneider T., Szidat S., Lloren R., Grosjean M. (2020). High-Resolution Historical Record of Plant Protection Product Deposition Documented by Target and Nontarget Trend Analysis in a Swiss Lake under Anthropogenic Pressure. Environ. Sci. Technol..

[74] Jiménez-Moreno G., Anderson R.S., Shuman B., Yackulic E. (2019). Forest and lake dynamics in response to temperature, North American monsoon and ENSO variability during the Holocene in Colorado (USA). Quat. Sci. Rev..

[75] Pedrotta T., Gobet E., Schwörer C., Beffa G., Butz C., Henne P.D., Morales-Molino C., Pasta S., van Leeuwen J.F.N., Vogel H. (2021). 8,000 years of climate, vegetation, fire and land-use dynamics in the thermo-mediterranean vegetation belt of northern Sardinia (Italy). Veg. Hist. Archaeobotany.

[76] Żarczyński M., Tylmann W., Goslar T. (2018). Multiple varve chronologies for the last 2000 years from the sediments of Lake Żabińskie (northeastern Poland)—Comparison of strategies for varve counting and uncertainty estimations. Quat. Geochronol..

[77] Fritz S.C., Anderson N.J. (2013). The relative influences of climate and catchment processes on Holocene lake development in glaciated regions. J. Paleolimnol..

[78] Heiri O., Ilyashuk B., Millet L., Samartin S., Lotter A.F. (2014). Stacking of discontinuous regional palaeoclimate records: Chironomid-based summer temperatures from the Alpine region. Holocene.

[79] Wilhelm B., Rapuc W., Amann B., Anselmetti F.S., Arnaud F., Blanchet J., Brauer A., Czymzik M., Giguet-Covex C., Gilli A. (2022). Impact of warmer climate periods on flood hazard in the European Alps. Nat. Geosci..

[80] Lowe D.J. (2011). Tephrochronology and its application: A review. Quat. Geochronol..

[81] Grimm E.C. (1987). Coniss: A Fortran 77 program for stratigraphically constrained cluster analysis by the method of incremental sum of squares. Comput. Geosci..

[82] Henares S., Donselaar M.E., Bloemsma M., Tjallingii R., De Wijn B., Weltje G. (2019). Quantitative integration of sedimentological core descriptions and petrophysical data using high-resolution XRF core scans. Mar. Pet. Geol..

[83] Croudace I.W., Rothwell R.G., Croudace I.W., Rothwell R.G. (2015). Future Developments and Innovations in High-Resolution Core Scanning. Micro-XRF Studies of Sediment Cores.

[84] Zander P.D. (2021). The Varved Sediments of Lake Żabińskie, Poland as a High-Resolution Archive of Environmental Change. Ph.D. Thesis.

[85] Grosjean M., Amann B., Butz C., Rein B., Tylmann W. (2014). Hyperspectral imaging: A novel, non-destructive method for investigating sub-annual sediment structures and composition. Past Glob. Chang. Mag..

[86] Thrane J.-E., Kyle M., Striebel M., Haande S., Grung M., Rohrlack T., Andersen T. (2015). Spectrophotometric Analysis of Pigments: A Critical Assessment of a High-Throughput Method for Analysis of Algal Pigment Mixtures by Spectral Deconvolution. PLoS ONE.

[87] Sanchini A., Grosjean M. (2020). Quantification of chlorophyll a, chlorophyll b and pheopigments a in lake sediments through deconvolution of bulk UV–VIS absorption spectra. J. Paleolimnol..

